# Supplementary material for: The relationship between night-time light and socioeconomic factors in China and India
Source: PLoS One. 2022 Jan 13;17(1):e0262503. doi: 10.1371/journal.pone.0262503 (PMC8758086; doi:10.1371/journal.pone.0262503)
Supplement: Attachment — Submitted filename: Response to editors.docx [file pone.0262503.s002.docx]

Dear editor

Thank you very much for your careful review of the manuscript. To your two questions, we give the following answers:

1. We have changed the financial disclosure in the online system, but because of some reasons, the change was not successful. We deleted the National Natural Science Foundation of China (No. 42071314) and the National Natural Science Foundation of China under Grant (No. 41771369), and replaced them with the General Project of National Social Science Fund of China (17BJY053). Therefore, our final financial disclosure is the General Project of National Social Science Fund of China (17BJY053). Professor Zhou Tao received a salary from the General Project of National Social Science Fund of China (17BJY053).
2. We can make all minimal data underlying the findings described in our manuscript fully available without restriction at the time of publication. We just selected it by mistake the second time we submitted the manuscript. In fact, the data used in the manuscript is very basic statistical data, and does not involve any ethical or legal issues.

If there is any question in the revised version of our manuscript, please feel free to contact us and we will make our best effort to meet your requirements. We sincerely thank you for consideration of our paper for publication in PLOS ONE.

Sincerely yours,

Han Guhuai
