# Supplementary material for: The relationship between night-time light and socioeconomic factors in China and India
Source: PLoS One. 2022 Jan 13;17(1):e0262503. doi: 10.1371/journal.pone.0262503 (PMC8758086; doi:10.1371/journal.pone.0262503)
Supplement: Attachment — Submitted filename: Response to Reviewers.docx [file pone.0262503.s003.docx]

Title:The relationship between night-time light and socioeconomic factors in China and India

Response to editors:

Dear editors:

Thank you for checking my manuscript carefully. To your question about reference list, we give the following answers:

We checked the reference list and found that some newly published Chinese papers we cited may not have been included in the English database. We have replaced this part of references or changed the citation name, such as references 3, 27,28,29,31, and 34. According to the requirements of reviewers, we added references 50 and 54. For the modification of other parts of the manuscript, please see the Response to reviewers.

If there is any question in the revised version of our manuscript, please feel free to contact us and we will make our best effort to meet your requirements. We sincerely thank you for consideration of our paper for publication in PLOS ONE..

Sincerely yours,

HAN Guhuai

Response to Reviewer Comments:

Dear Reviewer:

Thank you very much for your careful review of my manuscript, and points out some omissions in the manuscript. This has greatly helped this manuscript to have a chance to become a paper. We have carefully considered your all opinions, and made some changes in manuscript. The revisions use the "Track Changes" in the revised manuscript. Detail of the revisions are listed as follows.

1.Abstract-Line 24, GDP (gross domestic product)? Maybe gross domestic product (GDP)? Please confirm it

Thank you for your careful inspection. We have corrected this mistake in the manuscript.

2. Figure 1, is not clear enough. Please added national boundaries to make it clearer.

Thank you for your suggestion. We have added national boundaries to Figure 1 in our manuscript.

3. Line 143, CO2, please modify it and check the manuscript carefully.

I am sorry for my carelessness. We have checked all CO2 in the manuscript and changed it to CO_2_.

4. Discussion looks good. However, the author did not discuss the limitation of this study. Furthermore, some important relevant references should be cited as follow.

Thank you for your approval of the discussion section. In fact, we also talked about some limitations of the paper in the discussion and conclusion, such as the lack of research on spatial aggregation, factors, models and other issues, but they are scattered in the text and may be imperceptible. Now at the end of the discussion, we have added some new understanding of the limitations of this article.

The references you recommended are very valuable, especially the articles on the spatial distribution and time evolution of population and buildings. In fact, there is also a discussion on this issue in the manuscript. We have added these references such as 50 and 54. Several other articles about urban thermal effect are not directly involved in this article, so we have not quoted them.

If there is any question in the revised version of our manuscript, please feel free to contact us and we will make our best effort to meet your requirements. We sincerely thank you for consideration of our paper for publication in PLOS ONE.

Sincerely yours,

HAN Guhuai
