# Supplementary material for: The relationship between night-time light and socioeconomic factors in China and India
Source: PLoS One. 2022 Jan 13;17(1):e0262503. doi: 10.1371/journal.pone.0262503 (PMC8758086; doi:10.1371/journal.pone.0262503)
Supplement: Attachment — Submitted filename: Response to Reviewers.docx [file pone.0262503.s004.docx]

Title:The relationship between night-time light and socioeconomic factors in China and India

Response to editors:

Dear editors:

Thank you for checking my manuscript carefully. To your question about reference list, we give the following answers:

We checked the reference list and found that some newly published Chinese papers we cited may not have been included in the English database. We have replaced this part of references or changed the citation name, such as references 3, 27,28,29,31, and 34. According to the requirements of reviewers, we added references 50 and 54. For the modification of other parts of the manuscript, please see the Response to reviewers.

If there is any question in the revised version of our manuscript, please feel free to contact us and we will make our best effort to meet your requirements. We sincerely thank you for consideration of our paper for publication in PLOS ONE.

Sincerely yours,

HAN Guhuai

Response to Reviewer Comments:

Dear Reviewer:

Thank you very much for your careful review of my manuscript, and points out some omissions in the manuscript. This has greatly helped this manuscript to have a chance to become a paper. We have carefully considered your all opinions, and made some changes in manuscript. The revisions use the "Track Changes" in the revised manuscript. Detail of the revisions are listed as follows.

1. I found that the author order has changed, please explain it.

All the listed authors have made great contributions to this paper. The order of authors was adjusted a long time ago, which was decided by contributions and funds. The last manuscript we revised didn't change the order of authors, only Dr. Sun's author affiliation. Dr. Sun is a doctoral student who graduated from Peking University and is now teaching at Dalian Maritime University. So we added his author affiliation 3.

2. Table 1 mentioned GDP and population, and it was derived from RESDC and RBI. The resolution of GDP and population in China is 1km, what is the resolution of it in India? In other words, if raster data is not needed, why not use statistics data?

Thank you very much for your careful consideration of our statistical process, this problem is actually a difficulty that we have faced in the statistical process.

We only processed the state-level data in India (compared with the provincial level in China), so we directly used the statistical table data divided by administrative region. For China, in addition to the provincial level, we also process county-level data, so the resolution of statistical data is required to be higher. There are more than 2,800 county-level administrative regions in China, but China's Bureau of Statistics does not provide a unified data table, so it would be a lot of work to search each county. However, the Resource and Environmental Science Data Center of Chinese Academy of Sciences([www.resdc.cn](http://www.resdc.cn)) has provided 1km grid data of GDP and population, and this raster data is also made according to the county-level and township-level data of Chinese statistics, which is authoritative. Therefore, it is much more convenient to use raster data in China, and it is also conducive to some possible subsequent spatial relationship analysis or more detailed work.

3. There are many mistakes, please check it carefully.

Line 160 - First, we converted the carbon emission NC format into TIF format with GDAL ‘.’ is missing.

Table title is missing, e.g. Line 254, 293 and 296.

Line 268 - R2of ?

I am sorry for our carelessness. We have changed the problem you mentioned, and checked the similar problems in the full text. Since the two tables belong to the same multiple regression, I added subheadings to them. Line 268 - *R*^2^ are various regression *R*^2^of India (Fig 7).

4. Fig 2 is incomplete, please modify it.

Thank you for your suggestion. We have modified Figure 2 to make it correspond to the method in the paper.

5. Fig 3 - p value should be added in it. Please check it.

Thank you for your reminder. The analysis of this article mainly focuses on regression coefficient, *R*^2^ and MRE, so only regression coefficient, *R*^2^ and MRE are put in all the figures. We think that P value or F value can only be mentioned in the text or table to represent the persuasiveness of the data. After your reminder, we found that the p value was wrongly written when copied from SPSS to the text. It should be less than 0.01 instead of 0.00. We have made a comprehensive revision.

6. Line 237 and 191 - where adjusted R2? Please check it.

In multiple regression, there will be adjusted *R*^2^ affected by the number of dependent variables. Therefore, in order to prevent the negative effects of incorrect dependent variables, we recorded *R*^2^ and adjusted *R*^2^ for comparison. There are some punctuation errors here, and we have made unified changes.

If there is any question in the revised version of our manuscript, please feel free to contact us and we will make our best effort to meet your requirements. We sincerely thank you for consideration of our paper for publication in PLOS ONE.

Sincerely yours,

HAN Guhuai
